# Unveiling microbial worlds: exploring viral metagenomics among waste pickers at Latin America’s largest dumpsite

**DOI:** 10.1590/S1678-9946202466049

**Published:** 2024-08-26

**Authors:** Vanessa Resende Nogueira Cruvinel, Eneas de Carvalho, Daiani Cristina Cilião Alves, Carla Pintas Marques, Rafael dos Santos Bezerra, Marta Giovanetti, Sandra Coccuzzo Sampaio, Maria Carolina Elias, Wildo Navegantes de Araújo, Rodrigo Haddad, Svetoslav Nanev Slavov

**Affiliations:** 1 Universidade de Brasília, Faculdade de Ceilândia, Brasília, Distrito Federal, Brazil; 2 Instituto Butantan, Laboratório de Bacteriologia, São Paulo, São Paulo, Brazil;; 3 Centro Universitário Unieuro, Brasília, Distrito Federal, Brazil; 4 Universidade de São Paulo, Faculdade de Medicina de Ribeirão Preto, Hemocentro de Ribeirão Preto, Ribeirão Preto, São Paulo, Brazil; 5 Università Campus Bio-Medicio di Roma, Dipartimento di Scienze e Tecnologie per l´Uomo e l´Ambiente, Roma, Italy; 6 Fundação Oswaldo Cruz, Instituto Rene Rachou, Belo Horizonte, MG, Brazil; 7 Climate Amplified Diseases and Epidemics, Rio de Janeiro, Rio de Janeiro, Brazil; 8 Instituto Butantan, Centro de Vigilância Viral e Avaliação Sorológica, São Paulo, São Paulo, Brazil; 9 Universidade de Brasília, Núcleo de Medicina Tropical, Brasília, Distrito Federal, Brazil

**Keywords:** Waste pickers, Metagenomics, Infectious diseases Viral profile, Surveillance, Brazil

## Abstract

Waste pickers constitute a marginalized demographic engaged in the collection of refuse, facing considerable occupational hazards that heighten their susceptibility to contract infectious diseases. Moreover, waste pickers contend with societal stigmatization and encounter barriers to accessing healthcare services. To explore the viral profile of waste pickers potentially linked to their occupational environment, we conducted a metagenomic analysis on 120 plasma specimens sampled from individuals employed at the Cidade Estrutural dumpsite in Brasilia city, Brazil. In total, 60 blood donors served as a comparative control group. Specimens were pooled and subjected to Illumina NextSeq 2000 sequencing. Viral abundance among waste pickers revealed the presence of significant pathogens, including HIV, HCV, and Chikungunya, which were not detected in the control group. Additionally, elevated levels of anelloviruses and Human pegivirus-1 were noted, with a comparable incidence in the control group. These findings underscore the utility of metagenomics in identifying clinically relevant viral agents within underserved populations. The implications of this study extend to informing public health policies aimed at surveilling infectious diseases among individuals facing socioeconomic disparities and limited access to healthcare resources.

## INTRODUCTION

In Brazil, an estimated population of 200,000–800,000 individuals engage in waste picking, positioning the country among the global leaders in terms of absolute numbers within this social cohort^1^. Waste pickers typically operate independently, scavenging for recyclable materials with economic value. These individuals endure arduous working conditions characterized by physical hazards such as slips and falls, exposure to biological risks including infectious diseases, encounters with chemical substances leading to potential explosions, burns, and toxic exposures, as well as ergonomic challenges such as extreme heat and rainfall^1,2^. Moreover, waste pickers face social stigmatization, encounter barriers or complete absence of access to healthcare services, and lack fundamental sanitary provisions. Consequently, waste pickers may exhibit heightened rates of morbidity related to respiratory, ocular, gastrointestinal, neurological, and oncological ailments^2^.

The acquisition of infectious diseases is one of the main health hazards faced by waste pickers. Via external or internal injuries and contact with contaminated materials—such as hypodermic needles, surgical items, or laboratory glassware—waste pickers are susceptible to parenterally-transmitted viral infections, including HCV, HBV, and HIV^3^. Conversely, open-air dumpsites, particularly those located in tropical regions, provide conducive environments for the proliferation of arthropod vectors, thereby heightening the risk of waste pickers contracting arthropod-borne diseases, as evidenced in documented cases^4,5^. Moreover, waste pickers operating within open-air dumpsites may serve as fertile grounds for the emergence or re-emergence of infectious diseases, thus bearing significant implications for human health.

The diversity of viral infections that waste-pickers may encounter is extremely high, making it impractical to test for all these infectious agents. A suitable approach to evaluate the total genomic abundance and, in this context, the viral communities in clinical samples is viral metagenomics (the characterization of all viral sequences in a given sample). This methodology, which is based on next-generation sequencing (NGS), does not rely on diagnostic reactions or cell culturing, making it particularly suitable for describing emerging or unsuspected viruses^6^.

To assess the viral composition among waste pickers, we conducted a viral metagenomic survey on plasma samples collected from waste pickers operating at the largest open-air dumpsite in Latin America, namely Cidade Estrutural, situated in the Brasilia city, Federal District, Brazil. This site was formerly recognized as the second largest dumpsite globally until its official closure in 2018 by the Brazilian government. Spanning an area of 200 hectares and annually receiving 887,220 tonnes of refuse, it held significant prominence. By applying metagenomics to the plasma samples obtained from this cohort, we aimed to uncover the presence of previously undetected or even novel viral agents and elucidate the burden of viral diseases within this vulnerable population. The findings of this study hold substantial importance for enhancing public health policies aimed at addressing the needs and managing the health concerns of vulnerable populations, both in Brazil and worldwide.

## MATERIALS AND METHODS

### Plasma samples and study area

The study received ethical approval from the Research Ethics Committee of the Faculty of Medicine, University of Brasilia (CEP-FM/UnB, CAAE 39.892.420.7.1001.5558 and CAAE 40,557,020.6.3001.5553), as well as from the Fundacao de Ensino e Pesquisa em Ciencias da Saude (FEPECS/SES/DF, CAAE 40.557.020.6.3001.5553). All participants signed an informed consent form before being included in the study.

A total of 120 plasma samples were included in this study, collected from waste pickers from June to October 2017 at the Cidade Estrutural, an open-air dumpsite located in the Federal District of Brazil, Brasilia city, before its official deactivation. The Cidade Estrutural administrative region is home to approximately 40,000 residents, with around 4,000 individuals working as waste pickers. This region also exhibits the lowest human development index (HDI) within the Federal District. The natural boundaries of the Cidade Estrutural include the Brasilia National Park, which comprises tropical savanna. For further details on the Cidade Estrutural dumpsite, please refer to Supplementary Figure S1

As a control group for this study, plasma samples from 60 blood donors were obtained from the Blood Center of Ribeirao Preto city, located in the Southeastern Brazil. The inclusion of a control group from a different geographic region was based on the assumption that the blood virome of donors is relatively stable as observed elsewhere^7-9^. Besides, this stability can be attributed to specific characteristics of the blood donor recruitment such as regular tests for mandatory infectious diseases conducted by the blood banks and the standardized selection process during blood donation interview, which is consistent across the entire Brazilian territory. Following blood collection, plasma was promptly separated from the cellular component via low-speed centrifugation (3,000 rpm for 5 minutes) and stored at −80 °C until metagenomics analysis.

### Pre-preparation of plasma samples, nucleic acid extraction, and Illumina sequencing

Initially, 600 μL of individual plasma underwent pre-treatment with Turbo DNase (ThermoFisher Scientific) to remove host/bacterial DNA before extraction. Following DNase inactivation, eight plasma samples were assembled into pools. Pooling of samples was implemented to mitigate sequencing costs and enable testing of a larger sample size via metagenomics. Nucleic acids were extracted from the total pool volume using the High Pure Viral Nucleic Acid Large Volume Kit (Roche), with minor adaptations, including the incorporation of GenElute^®^ Linear Polyacrylamide carrier (LPA) (Merck) for nucleic acid concentration and isopropanol for precipitation. Post-extraction, nucleic acids were eluted in 50 µL of nuclease-free water preheated to 70 °C. Then, 5 µL of the extracted nucleic acids underwent reverse transcription using the Superscript III First-Strand Synthesis System (ThermoFisher Scientific). Subsequently, cDNA amplification was carried out using the QuantiTect Whole Transcriptome Kit (QIAGEN). Sequence libraries were prepared using the DNA Prep Library Preparation Kit (Illumina) and Nextera DNA CD Indexes, using 500 ng of amplified transcript for tagmentation. Pair-end sequencing of the dual-indexed libraries was performed on the Illumina NextSeq 2000 sequencing platform employing the NextSeq P3 flowcell (300 cycles) (Illumina), following the manufacturer’s instructions.

### Bioinformatic processing of the raw sequencing data and taxonomic classification of viral reads

The raw sequence data obtained underwent quality control analysis using the FastQC software (version 0.11.8, Babraham Institute, Cambridge, UK). Subsequently, trimming and adapter removal were conducted using the TrimGalore (version 0.6.6, Babraham Institute, Cambridge, UK) and Fastp (version 0.23.1, HaploX Biotechnology, Shenzhen, China) programs to select sequences exhibiting optimal quality and devoid of adapters. For metagenomic analysis, only reads with a quality score >30 were retained.

To infer the taxonomic classification of the virome, the Kraken software (version 2.0.8, Johns Hopkins University, Baltimore, MD, USA) was used along with the genomic minikraken2 database. Additionally, Kraken was employed to eliminate human, bacterial, and parasitic reads, which were subsequently excluded from further analysis. Viruses that do not induce human infections, as well as phages and artifacts, were manually excluded from the Kraken results as they did not align with the objectives of this study.

### Viral diversity analysis

The abundance of the viral communities was estimated based on the normalized number of read counts as previously described^10^. In addition, Kraken outputs were transformed into BIOM-format (Biological Observation Matrix) tables using the Kraken-BIOM (Biofrontiers Institute, University of Colorado, Boulder, CO, USA), and then, in the R programming language (version 2.1, Foundation of Statistical Computing, Viena, Austria), using the packages phyloseq (Department of Statistics, Stanford University, Stanford, CA, USA) and vegan (University of Helsinki, Helsinki, Finland) to estimate and plot the Shannon and Simpson indices of diversity and the rarefaction curves. Moreover, the ggplot2 (University of Auckland, Auckland, New Zealand) and heatmap2 (Boehringer-Ingelheim, Ingelheim am Rhein, Germany) packages were used to plot data.

## RESULTS

### Waste pickers demographics

In this study, a total of 120 plasma samples from waste pickers were included. The median age of the study cohort was 39.16 years (range: 19–66 years). Regarding gender distribution, most participants were women (n=86/120, 71.7%), while the remaining participants were men (n=34/120, 28.3%). All participants were engaged in waste collection as their primary occupation. The control group comprised blood donors with a gender distribution of 60% women and 40% men. The mean age of the control group was 38 years.

### Sequencing characteristics of the waste picker samples

Next-generation sequencing (NGS) conducted on pools of waste pickers yielded a satisfactory read count per pool, with a mean of 58,718,730.47 reads. Supplementary Table S1 summarizes the quantitative attributes of NGS subsequent to trimming and the removal of bacterial and human reads. The average number of viral reads per pool was 2,839 (0.005% of the total read count). Conversely, the controls exhibited an average read count of 5,654,522 per pool, with a mean number of viral reads at 27,339.5 (0.5%).

### Viral abundance

**Figure 1:**
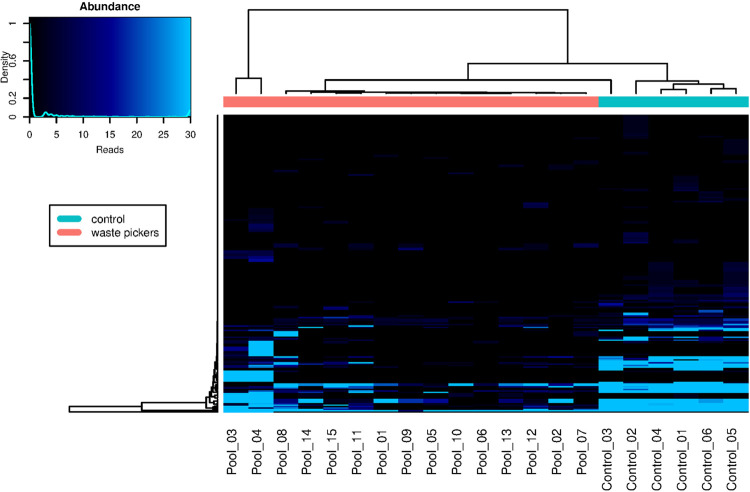
Heatmap displaying viral abundance based on the total number of viral reads obtained from each analyzed sample. The color scheme key (upper left) illustrates the correlation between colors and the number of reads. Hierarchical clustering is presented for both samples and viral species/genus, revealing the separation of the pools into three distinct clusters.

The heatmap depicted in Figure 1 illustrates the viral abundance by species, revealing a hierarchical clustering that delineates three distinct clusters encompassing both control and waste-picker samples. The largest cluster predominantly comprises samples from waste pickers, alongside one pool from the control group (Control_3). Another cluster exclusively comprises samples from the control group. Notably, two pool samples from waste pickers formed a separate cluster, possibly indicative of divergent viral diversity.

**Figure 2:**
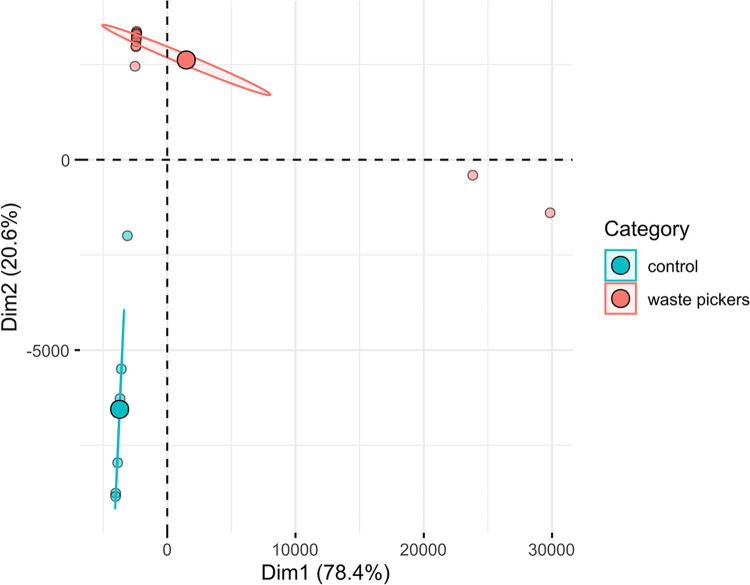
Principal component analysis (PCA) illustrating the separation of the studied samples. Individual samples are represented by small circles, whereas large circles denote the centroid for each group. The ellipse encompasses a 95% confidence interval.

**Figure 3:**
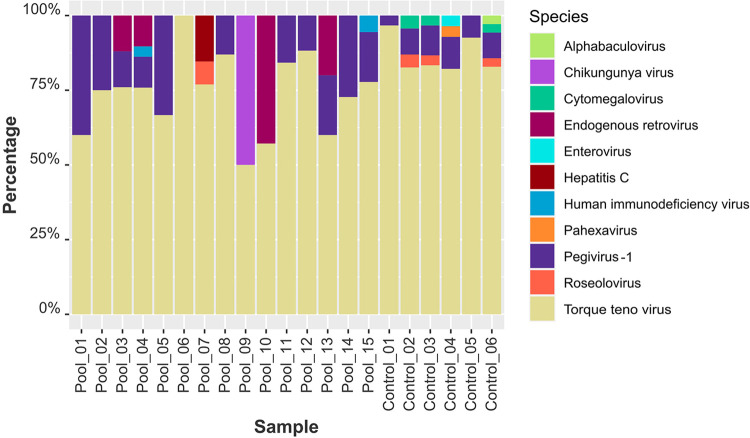
Relative abundance of human viruses (curated dataset) in waste pickers (identified as pools_1 to 15) and controls that represent pooled blood donor plasmas (control_01 to control_06). Notable findings include the identification of HCV reads in pool 3, HIV in pools 4 and 15, Chikungunya virus reads in pool 9, and Human Herpesvirus 6 (HHV-6) reads in pool 7. Moreover, across all pools, a high abundance of anelloviruses and human pegivirus-1 is observed, consistent with their stable presence in the human virome.

Moreover, principal component analysis (PCA) (Figure 2) corroborates this variability pattern. To delve deeper into the presence of human viruses among waste pickers, we scrutinized the total abundance of the analyzed virome. Across all samples, anelloviruses were abundantly present, consistent with their integral role in the human virome. This predominance was particularly pronounced in control samples. Notably, human pegivirus-1, another commensal virus, exhibited robust representation in waste-picker samples, with markedly higher read counts compared to healthy controls (Figure 3).

Moreover, the metagenomic analysis showed clinically significant human pathogens, including reads for HIV, HCV, Chikungunya virus (CHIKV), and Human herpesvirus-6 in several pools obtained from waste-pickers. In blood donors, we identified a low number of reads belonging to Human Cytomegalovirus (CMV) in three pools, as well as Rhinovirus group A reads in one pool, both presenting in low quantities. Figure 3 shows the normalized viral abundance of the curated dataset.

## DISCUSSION

In this study, we investigated the viral composition among waste pickers operating at the Cidade Estrutural dumpsite in Brasilia city, Federal District of Brazil. Until its official deactivation, this site was considered the largest dumpsite in Latin America and one of the largest globally. We hypothesized that the precarious working conditions and the proximity to natural biomes could potentially lead to a distinctive virome composition within the studied group compared to the general population. However, the main purpose of this study was to draw attention to the necessity for public policies focused on surveillance and management strategies tailored to vulnerable populations. By elucidating the viral landscape of waste pickers, we sought to contribute valuable insights to the development of targeted interventions aimed at safeguarding the health and well-being of these individuals.

The identification of clinically significant viruses, including HCV, HIV, HHV-6, and CHIKV, via viral metagenomic analysis of the plasma of waste pickers, underscores the pressing need for targeted interventions within this population. In contrast, the limited presence of pathogenic viruses in the control group highlights the distinct viral landscape encountered by waste pickers. The presence of parenterally-transmitted viruses such as HIV and HCV among waste pickers is a well-documented phenomenon in the literature, and the identification of positive samples is expected. Studies have reported HIV seroprevalence rates of 0.75% and HCV rates of 0.11% among waste pickers^3^. These sexually transmitted infections are often associated with low socioeconomic conditions, particularly in populations lacking adequate resources to comprehend their exposures and associated risks, thus hindering appropriate health behavior and treatment-seeking. Moreover, the occupational acquisition of these infections is a significant concern. HIV, HCV, and HBV can contaminate solid waste improperly deposited in dumpsites^3^. Consequently, there is an urgent need for further research to assess the occupational risks, such as injuries and cuts, among waste pickers for acquiring these infections. Such studies are essential for informing targeted interventions aimed at mitigating occupational risks and promoting the health and safety of waste pickers.

The detection of CHIKV reads in a pool obtained from waste pickers, alongside their absence in the control group, is significant and indicative of potential arboviral transmission within this population. Arboviruses, including CHIKV, are primarily transmitted by mosquito vectors, which thrive in dumpsites due to the favorable conditions for mosquito proliferation. Interestingly, a recent metagenomic study conducted in the vicinity of the dumpsite revealed several CHIKV infections in the saliva of tested patients^11^. This finding suggests that waste pickers may indeed face an increased risk of acquiring arboviral infections directly related to their occupation. Overall, these findings underscore the importance of implementing measures to mitigate the risk of arboviral infections among waste pickers, as well as addressing the broader health challenges faced by this vulnerable population. Efforts to improve sanitation, vector control, and occupational safety are imperative to safeguard the health and well-being of waste pickers and prevent the transmission of infectious diseases in dumpsite environments.

We identified few sequence reads belonging to human cytomegalovirus (CMV) in several pooled blood samples from donors. Due to the application of leukoreduction in blood donations, the risk of CMV transfusion-transmitted infection is considered invariably low. This risk generally results from infectious virus present during acute infection or reactivation in blood donors^12^. However, since we detected sequences of CMV rather than the infectious virus, it is difficult to draw any definitive conclusions about the impact of this finding among blood donors.

Despite the identification of clinically important viruses exclusively among waste pickers and their absence in blood donors, the principal component analysis did not exhibit a distinct division between the viromes of waste pickers and blood donors. While two pool samples from waste pickers clustered together, likely due to their divergent virus profiles, one pool sample from the control group was interspersed among pools obtained solely from waste pickers. We propose that the principal components of the commensal virome observed in healthy individuals, including waste pickers irrespective of their social status, such as anelloviruses and HPgV-1, are uniformly present throughout the Brazilian population. However, we did not conduct in-depth studies on the taxonomy of the identified commensal viruses. We believe that further specialized studies are needed to characterize the differences in the anellovirome/commensal virome in vulnerable populations. The uniformity of the virome suggests that, while there may be differences in the prevalence of pathogenic viruses, the foundational components of the virome remain consistent across diverse populations. This suggestion is supported by numerous studies evaluating the viromes of healthy individuals, which consistently demonstrate the ubiquitous presence of anelloviruses and HPgV-1 as integral components of the human virome^9,13^.

A limitation of our study was the exclusion of further steps in genomic assembly and detailed phylogenetic analysis of the detected viral agents. This limitation was addressed by the analysis based on reads mapping to a large database of viral genomes, reaching a reliable degree of taxonomic classification despite their size. Additionally, the samples were pooled, which means that assembled genomes in this case could result in chimeric sequences unsuitable for further analysis.

## CONCLUSION

In conclusion, this study employed viral metagenomics to explore the presence of emerging or previously undetected viruses within a marginalized population of waste pickers. Clinically significant viruses were identified, in contrast to the control group, suggesting a potential association with their occupational or low socioeconomic status. While this study delineates the qualitative structure of the virome composition in this population, further research is suggested to elucidate the cause-effect relationship between identified infections and working conditions. The findings of this study highlight the importance of monitoring specific infections in vulnerable populations, which could inform public health policies and practices. By considering social, cultural, and economic factors in conjunction with epidemiological data, policymakers can develop targeted interventions aimed at safeguarding the health and well-being of marginalized communities such as waste pickers.

## Data Availability

https://doi.org/10.48331/scielodata.LHBMY8
