# Supplementary material for: Unveiling microbial worlds: exploring viral metagenomics among waste pickers at Latin America’s largest dumpsite
Source: Rev Inst Med Trop Sao Paulo. 2024 Aug 26;66:e49. doi: 10.1590/S1678-9946202466049 (PMC11348794; doi:10.1590/S1678-9946202466049)
Supplement: Supplementary file 1 [file 1678-9946-rimtsp-66-S1678-9946202466049-suppl01.pdf]

REVISTA DO INSTITUTO DE MEDICINA TROPICAL DE SÃO PAULO

JOURNAL OF THE SÃO PAULO INSTITUTE OF TROPICAL MEDICINE

SUPPLEMENTARY MATERIAL

https://doi.org/10.48331/scielodata.LHBMY8

Unveiling microbial worlds: exploring viral metagenomics among waste pickers at Latin America's largest dumpsite

Vanessa Resende Nogueira Cruvinel[1], Eneas de Carvalho[2], Daiani Cristina Cilião Alves[3], Carla Pintas Marques[1], Rafael dos Santos Bezerra[4], Marta Giovanetti[5,6,7], Sandra Coccuzzo Sampaio[8], Maria Carolina Elias[8], Wildo Navegantes de Araújo[1,9], Rodrigo Haddad[1,9], Svetoslav Nanev Slavov[8]

[1]Universidade de Brasília, Faculdade de Ceilândia, Brasília, Distrito Federal, Brazil

[2]Instituto Butantan, Laboratório de Bacteriologia, São Paulo, São Paulo, Brazil;

[3]Centro Universitário Unieuro, Brasília, Distrito Federal, Brazil

[4]Universidade de São Paulo, Faculdade de Medicina de Ribeirão Preto, Hemocentro de Ribeirão Preto, Ribeirão Preto, São Paulo, Brazil

[5]Università Campus Bio-Medicio di Roma, Dipartimento di Scienze e Tecnologie per l´Uomo e l´Ambiente, Roma, Italy

[6]Fundação Oswaldo Cruz, Instituto Rene Rachou, Belo Horizonte, MG, Brazil

[7]Climate Amplified Diseases and Epidemics, Rio de Janeiro, Rio de Janeiro, Brazil

[8]Instituto Butantan, Centro de Vigilância Viral e Avaliação Sorológica, São Paulo, São Paulo, Brazil

[9]Universidade de Brasília, Núcleo de Medicina Tropical, Brasília, Distrito Federal, Brazil

Correspondence to: Svetoslav Nanev Slavov
Instituto Butantan, Centro de Vigilância Viral e Avaliação Sorológica, Avenida Vital Brasil, 1500, Butantã, CEP 05585-000, São Paulo, SP, Brazil
Tel: +55 11 2627-9734

E-mail:
svetoslav.slavov@fundacaobutantan.org.br

Received: 6 May 2024

Accepted: 18 June 2024

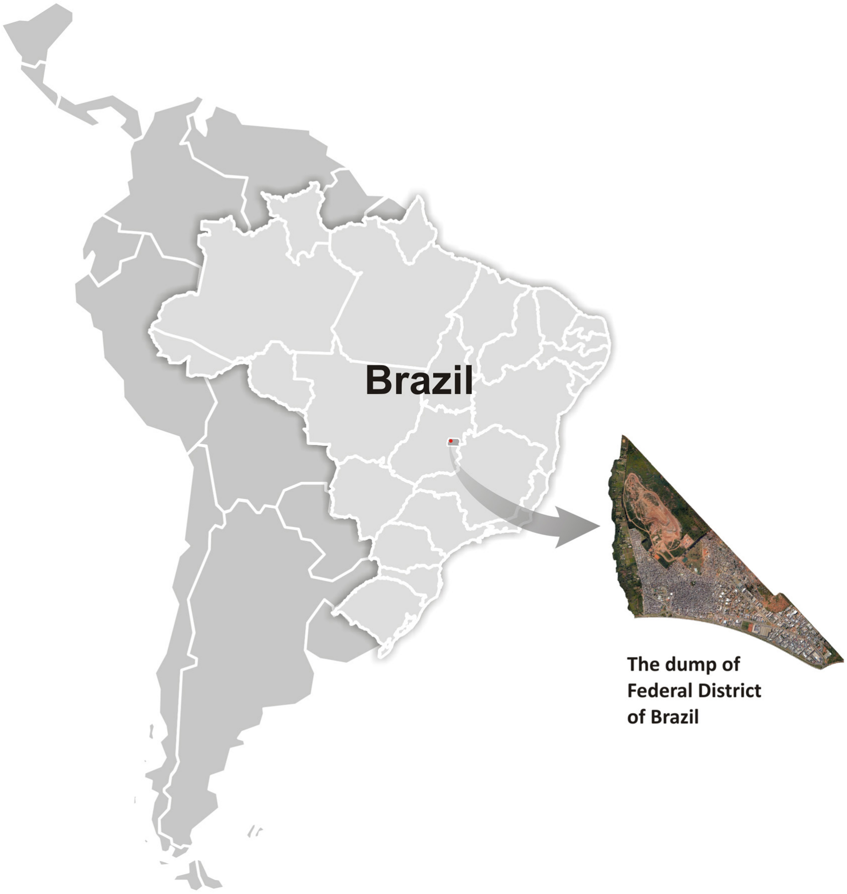

Supplementary Figure S1 - Spatial area under investigation: The study samples were obtained from waste pickers operating at the Cidade Estrutural open-air dumpsite, situated approximately 10 km from the administrative center of the Federal District of Brazil, in Brasilia city. Formerly recognized as the largest open-air dumpsite in Latin America and one of the largest globally, it was officially deactivated in 2018.

This is an open-access article distributed under the terms of the Creative Commons Attribution License.

Supplementary Table S1 - Sequencing data acquired from sample pools originating from waste pickers as well as from the control group comprised of blood donors.

Pool	Raw reads	Reads after trimming	Viral reads
WP1[a]	55,179,547	49,478,546	2,173
WP2	61,676,743	59,132,249	2,956
WP3	65,772,821	63,762,891	3,188
WP4	49,694,460	47,345,695	2,367
WP5	56,335,672	54,932,256	2,746
WP6	71,341,891	69,004,442	3,450
WP7	57,342,213	55,847,224	2,792
WP8	49,179,547	48,479,544	2,423
WP9	51,676,743	50,136,298	2,506
WP10	67,198,921	66,322,513	3,316
WP11	49,794,989	49,124,995	2,456
WP12	76,320,072	75,930,076	3,796
WP13	61,741,471	59,298,155	2,964
WP14	58,346,320	57,847,561	2,892
WP15	49,179,547	48,479,544	2,566
C12	5,567,102	4,847,954	26,054
C2	4,978,098	4,113,674	29,759
C3	5,716,292	7,630,252	23,055
C4	4,478,477	4,912,499	21,323
C5	5,643,987	3,593,007	21,689
C6	7,543,179	7,129,814	42,157

[a]Waste pickers pools; bControl group (blood donors) pools.
